# Comparative evaluation of two reconstructive methods following laparoscopic assisted subtotal gastrectomy in dogs

**DOI:** 10.1186/1756-0500-5-679

**Published:** 2012-12-11

**Authors:** Jalal Bakhtiari, Mahbobeh Abdi, Alireza R Khalaj, Farzad Asadi, Amir Niasari-Naslaji

**Affiliations:** 1 Faculty of Veterinary Medicine, University of Tehran, Tehran, Iran; 2 Department of Surgery, Faculty of Medicine, Shahed University, Tehran, Iran; 3 Minimally Invasive Surgery Research Centre, Rasool Akram Hospital, Tehran University of Medical Sciences, Tehran, Iran; 4 Department of Surgery and Radiology, Faculty of Veterinary Medicine, University of Tehran, P. O. Box: 14155-6453, Tehran, Iran

**Keywords:** Gastrectomy, Laparoscopy, Reconstruction, Dog

## Abstract

**Background:**

Laparoscopic gastrectomy is a new and technically challenging surgical procedure with potential benefit. The objective of this study was to investigate clinical and para-clinical consequences following Roux-en-Y and Jejunal Loop interposition reconstructive techniques for subtotal gastrectomy using laparoscopic assisted surgery.

**Results:**

Following resection of the stomach attachments through a laparoscopic approach, stomach was removed and reconstruction was performed with either standard Roux-en-Y (n = 5) or Jejunal Loop interposition (n = 5) methods. Weight changes were monitored on a daily basis and blood samples were collected on Days 0, 7 and 21 post surgery. A fecal sample was collected on Day 28 after surgery to evaluate fat content. One month post surgery, positive contrast radiography was conducted at 5, 10, 20, 40, 60 and 90 minutes after oral administration of barium sulfate, to evaluate the postoperative complications. There was a gradual decline in body weight in both experimental groups after surgery (P < 0.05). There was no difference in blood parameters at any time after surgery between the two methods (P > 0.05). Fecal fat content increased in the Roux-en-Y compared to the Jejunal loop interposition technique (P < 0.05). No major complications were found in radiographs and gastric emptying time was similar between the two groups (P > 0.05).

**Conclusion:**

Roux-en-Y and Jejunal loop interposition techniques might be considered as suitable approaches for reconstructing gastro-intestinal tract following gastrectomy in dogs. The results of this study warrant further investigation with a larger number of animals.

## Background

Several approaches of open surgeries including Roux en Y (gastrojejunostomy), Billroth I and jejunal loop interposition (jejunoduodenostomy) and Billroth II (gastrojejunal anastomosis) were used to reconstruct partial or total gastrectomy [1-4]. Comparing Roux en Y and Billroth II procedures, the Roux-en-Y approach showed less reflux symptoms and less chronic fundic atrophic gastritis [5]. Although Roux-en-Y is the most common method of reconstruction for total gastrectomy [1,6], it is associated with abdominal pain, nausea, vomiting, fullness and morbidity, as high as 30% [7-10]. Furthermore, due to the lack of food passage through the duodenum, the potential benefit of the duodenum for normal digestion is disrupted following Roux-en-Y reconstructive technique, [11,12]. On the contrary, in Jejunal loop interposition, the food passage through the duodenum was maintained [6-8,13]. Regardless, there were no differences between Roux-en-Y and Jejunal Loop interposition in prospective quality of life, and no complications in long term studies [14-16].

Laparoscopic assisted surgery has several advantages compared with open surgery including reduced blood loss, less postoperative pain, better oral intake, earlier bowel function recovery, shorter time of hospital stay and reduced risk of sepsis [17]. The laparoscopic assisted procedure is less invasive than conventional open gastrectomy. It leads to quick recovery of gastrointestinal functions and reduces pain [18]. More recently, we have demonstrated that laparoscopic assisted Roux-en-Y reconstructive technique had less post-operative complications compared to open surgery in dogs [19]. Laparoscopic assisted subtotal gastrectomy is a feasible and safe alternative approach compared with an open surgery [20,21]. In the present study, laparoscopic assisted subtotal gastrectomy was conducted using Roux-en-Y or Jejunal Loop interposition reconstructive techniques in dogs (n = 5 in each group).

## Methods

The present study received an approval by the Animal Ethics Committee of the Faculty of Veterinary Medicine, University of Tehran (BNS498/20.05.08). The experiment was conducted at Small Animal Veterinary Medicine Hospital, Faculty of Veterinary Medicine, University of Tehran. Ten healthy mixed-breed male dogs (26.5 ± 1.99 Kg of weight; 3.2 ± 0.29 years of age) were selected among homeless animals by permission from the animal shelter. All dogs received rabies vaccination and anti-parasitic agents (Praziquantel 5 mg/kg and piperazine 100 mg/kg, p. o). Prior to the surgery, the experimental dogs received a balanced diet with free access to water. Following surgery, food and water restriction were implemented while dogs received Ringer-Lactate serum for three days subsequent with the balanced soft and/or syrupy diet supplemented with minerals and vitamins.

**Figure 1:**
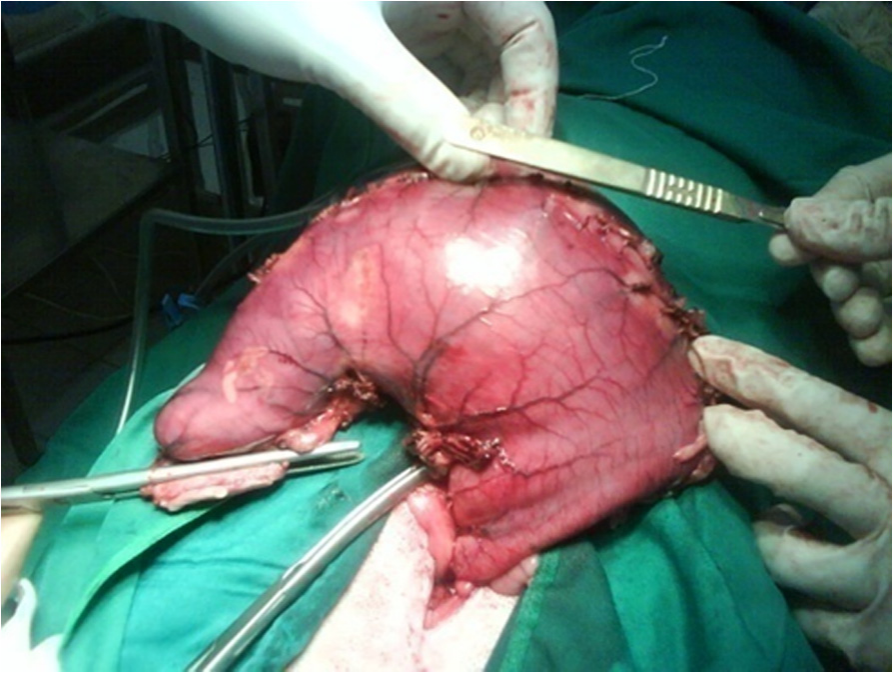
The stomach was removed from mini-laparotomy incision.

**Figure 2:**
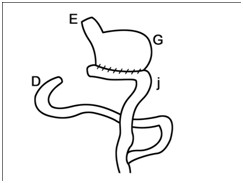
Roux-en-Y procedure.

**Figure 3:**
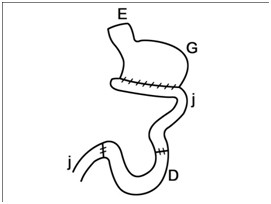
Jejunal Loop Interposition procedure.

Under general anesthesia (Acepromazine 0.04 mg/kg, IM; Tiopental-sodium 10 mg/kg, IV; Halothane, 1–1.5%) laparoscopic assisted gastrectomy was performed with three laparoscopic portals. The abdomen was insufflated with carbon dioxide (P = 14 mmHg). Following insertion of the first trocar (10 mm) at the umbilicus, the laparoscopic telescope (0 degree; 10 mm) was introduced into the abdominal cavity. The other two trocars (5 mm) were placed in 1/3 upper left and right midline. Gastric vessels were clipped and cut by clip-applicator and bipolar cautery, respectively. The gastroepiploic vessels and its branches were resected after coagulation with bipolar electrocuater forceps. After resecting stomach attachments, a 5 cm incision was performed on the place of the first laparoscopic trocar and four fifth of the distal stomach was removed (Figure 1). After gasterectomy, dogs were assigned randomly into two reconstructive techniques (n = 5 in each group). In Roux-en-Y group, the cutting edge of duodenum was closed with two layer inverting suture by hand sewing. Then the jejunum was cut from the 20 cm distal of the Treitz ligament. The distal part of the cutting edge of the jejunum was anastomosed to the remaining part of the stomach and the proximal part was anastomosed to the rest of the jejunum using the end to side pattern suturing by hand sewing (Figure 2). In the Jejunal Loop interposition group, the reconstructive technique was performed with the resection of 20 cm length of jejunum from the 20 cm distal to Treitz ligament. Then, two ends to side anastomosis were applied between the remaining part of the stomach and proximal part of the jejunum and between the distal part of the jejunum and duodenum (Figure 3). Finally the abdomen was lavaged and closed in a routine manner. The food restriction was continued for three days after surgery. Experimental dogs were weighed daily before and up to 30 days after surgery.

Blood samples were collected on Days 0, 7 and 21 after surgery. Serum was extracted and stored at −20°C until assayed. Urea concentration was measured using diacetylmonoxime reagent. Creatinine concentration was measured by the Jaffe reaction. Aspartate aminotransferase activity was measured by the direct combination of onalacetic acid with dinitrophenylhydraline and by the color change in an alkaline solution. Alkaline phosphatase activity was measured with p-nitrophenyl phosphate as a substrate. Total protein concentration was measured by the Biuret method. Triglyceride concentration was measured by the glycerol-phosphate onidase p-aminophenazone method. Cholesterol was measured by the cholesterol onidase p-aminophenazone method. Glucose concentration was measured by glucose onidase–aminophenazone method. Hexose concentration was measured by o-toluidine reagent. Sodium and potassium concentrations were determined by flame photometry. Fecal samples were taken on Day 28 after surgery and the quality of stool fat was estimated using Sudan black ΙΙΙ method. All materials and kits of biochemical tests were purchased from Ziest Chemie Diagnostics, Iran.

One month after surgery, 6 serial positive contrast radiographs were taken at 5, 10, 20, 40, 60 and 90 minutes after oral administration of barium sulfate (Daroupakhsh pharmaceutical, Iran) in order to determine the postoperative complications including leakage from the sites of anastomosis, narrowing, strictures or obstruction on the anastomotic sites, gastric emptying time and other abnormalities involving the small intestine.

Data were analyzed using GLM procedure with repeated measures included in the model. Single point measurements were tested using t-student test and Kruskal-Wallis test.

## Results

All dogs recovered from anesthesia with no complications during the surgery and recovery. There was no evidence of wound infection or dehiscence in patients. Dogs started to eat on Day 4 after surgery without any sign of regurgitation. No serious postoperative complications were observed. There were no signs of premature vasomotor disturbances, such as dizziness, faintness, weakness and alimentary disorders. One dog in Roux-en-Y group showed nausea, regurgitation and vomiting and received conservative therapy.

**Figure 4:**
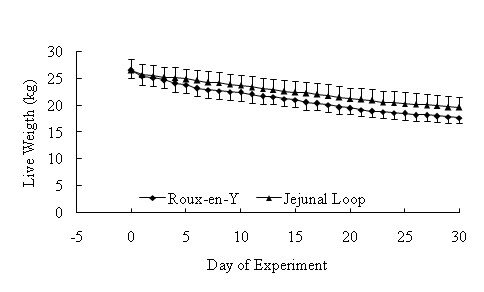
Body weight reduction until one month after surgery in both groups.

Body weights before the operation were similar between groups (Roux-en-Y: 26.5 ± 2.04 kg; Jejunal loop interposition: 26.5 ± 1.5 kg; P > 0.05). There was gradual reduction in body weight after surgery in both groups (Figure 4). Body weight one month after surgery reached 17.7 ± 1.98 kg and 19.6 ± 1.09 kg in Roux-en-Y and Jejunal loop interposition, respectively. In this study, 30 days after surgery, all experimental animals have shown significant weight loss but the difference between two groups was not significant. Fecal fat was observed in animals after Roux-en-Y operation but not after Jejunal Loop interposition.

Blood parameters, including Glucose, Hexose, TC, TG, TG/TC, Total protein, Albumin, Globulin, Urea, BUN, Creatinin, BUN/Creatinin, Fe, SGOT, SGPT, ALP. CL, Na and K were not different between experimental groups (Table 1; P > 0.05).

**Table 1:** Biochemical parameters of two experimental groups (Roux-en-Y and Jejunal Loop interposition using assisted laparoscopy) on Day 1, 7 and 21 after subtotal gastrectomy in dogs

**Parameters**	**Groups**	**Roux-en-Y**			**Jejunal loop interposition**		
	**Days**	**1**	**7**	**21**	**1**	**7**	**21**
Glucose(mm/dl)		4.1 ± 1.54	3.7 ± 0.27	3.6 ± 0.49	4.9 ± 0.66	4.7 ± 0.63	4.9 ± 0.52
Hexose(mg/dl)		70.7 ± 34.24	58.9 ± 4.47	57.1 ± 11.05	104.6 ± 15.09	87.9 ± 9.35	96.9 ± 7.98
Total Cholestrol (mm/l)		2.9 ± 0.32	3.1 ± 0.83	1.8 ± 0.41	2.8 ± 0.32	1.6 ± 0.11	1.5 ± 0.05
Three Glyceride(mm/l)		0.5 ± 0.12	0.4 ± 0.07	0.3 ± 0.07	0.4 ± 0.14	0.3 ± 0.06	0.4 ± 0.09
Tg/Tc		0.4 ± 0.06	0.4 ± 0.24	0.5 ± 0.04	0.4 ± 0.16	0.5 ± 0.11	0.6 ± 0.15
Total Protein(g/dl)		8.7 ± 1.07	6.9 ± 0.37	7.4 ± 0.34	6.7 ± 0.31	5.9 ± 0.22	7.1 ± 0.68
Albumin(mg/dl)		5.9 ± 0.52	5.6 ± 0.14	5.1 ± 0.23	5.6 ± 0.32	5.3 ± 0.35	5.2 ± 0.29
Globulin(mg/dl)		2.7 ± 0.58	1.3 ± 0.32	2.3 ± 0.41	1.1 ± 0.12	0.5 ± 0.12	1.8 ± 0.82
Urea(mm/l)		6.5 ± 0.72	4.5 ± 0.56	5.2 ± 0.27	5.8 ± 0.91	5.8 ± 1.37	4.7 ± 0.75
BUN(mm/l)		6.6 ± 0.72	4.5 ± 0.56	5.2 ± 0.27	5.8 ± 0.91	5.8 ± 1.37	4.7 ± 0.75
Creatinine(mg/dl)		3.4 ± 0.62	2.3 ± 0.11	2.6 ± 0.14	2.6 ± 0.38	2.7 ± 0.38	3.2 ± 0.36
BUN/Creatinine		5.6 ± 0.86	5.5 ± 0.86	5.5 ± 0.15	6.1 ± 0.24	5.8 ± 1.16	4.2 ± 1.02
Fe(um/l)		40.4 ± 11.72	15.7 ± 6.86	16.3 ± 4.42	15.01 ± 0.88	28.2 ± 2.51	15.9 ± 2.01
SGOT(Iu/l)		234 ± 71.02	38.6 ± 26.52	54 ± 14	104.3 ± 4.33	90.5 ± 24.66	96.9 ± 58.74
SGPT(Iu/l)		10.5 ± 5.37	3.4 ± 2.32	4.9 ± 1.04	20.2 ± 11.83	3.7 ± 1.41	9.2 ± 5.23
ALP(u/l)		106.8 ± 64.08	244.3 ± 125.28	74.3 ± 29.02	102.9 ± 75.37	70.4 ± 2.45	111.4 ± 79.46
Cl(mmol/l)		137.1 ± 2.97	131.1 ± 5.95	134.7 ± 5.67	128.1 ± 4.24	138.2 ± 2.59	128.1 ± 2.97
Na(mmol/l)		159.3 ± 6.33	160 ± 4.93	156 ± 1.52	160 ± 4.16	159.3 ± 6.64	162.3 ± 2.33
K(mmol/l)		5.8 ± 0.55	6.3 ± 0.28	6.03 ± 0.14	6.1 ± 0.34	5.9 ± 0.44	6.3 ± 0.26

**Figure 5:**
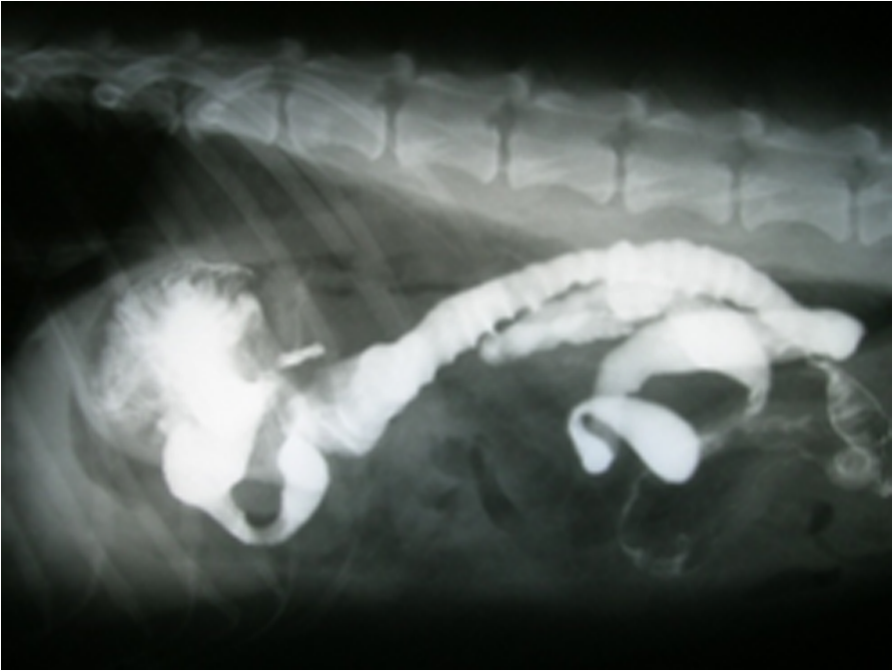
Positive contrast radiographs showing more jejunal movement than normal without any signs of leakage, strictures and other abnormalities.

Radiographic findings, conducted for 3 days after surgery, did not reveal any signs of anatomic abnormality, organ displacement, anastomotic leakage and narrowing or obstruction at the sites of the anastomosis. One month after surgery, radiographic findings did not show any signs of leakage or strictures at the anastomotic sites (Figure 5). Slight abnormal jejunal movements were observed in 2 dogs in Roux-en-Y and one dog in Jejunal loop interposition. Gastric emptying time was 89 ± 7.4 minutes in Roux-en-Y and 86 ± 4.1 minutes in Jejunal loop interposition (P > 0.05). There was no sign of narrowing, stricture or obstruction in the anastomotic sites.

## Discussion

Present study was conducted to compare Roux-en-Y and Jejunal Loop interposition reconstructive techniques for subtotal gastrectomy using laparoscopic assisted surgery in dog. All dogs recovered following operation without any particular complications. This could be due to the lesser inflammatory response of the gut and lesser bacterial and endotoxin release occurring following laparoscopic assisted gasterectomy [17].

The alterations in body weights were similar before and after operation between experimental groups. One month after surgery, animals in both groups have shown significant weight loss. Similarly, in most studies, significant body weight loss after gastrectomy was noticed [1,2,7,22-24]. In one study with ten patients, the average weight loss was 25 kg [23]. In another study, 24% body weight loss was noted in a group of 16 patients and only one-third of patients were able to achieve their ideal body weight after surgery [25]. Although the significant weight loss is a consequence of gastric resection, the cause of the malnutrition is still controversial. Malabsorption due to bacterial overgrowth, small intestinal mucosal lesions, pancreatic enzymatic deficiency and decrease of food passing time may be considered as part causes of malnutrition following gastric resection [26-28]. Insufficient caloric intake due to loss of appetite, intestinal motility alteration and early satiety may be also considered as other causes of malnutrition following gastrectomy [23,28,29].

In the present study, fecal fat was observed in animals after Roux-en-Y operation but not after Jejunal Loop interposition. Previously, it was reported that esophagojejunostomy did not affect fecal fat; while, fecal fat was higher in Roux-en-Y esophagojejunostomy group than esophagojejunoduodenostomy group [28]. The absorption of carbohydrate in the GI tract is high after gastrectomy, whether the duodenum was bypassed or not. In case of normal duodenal food passage, the rate of absorption for protein and fat after operation were 85% and 93%, respectively; whereas, this was slightly lower in duodenal bypass cases [30]. This illustrates the importance of the duodenal passing role in food absorption, especially for fat [28].

Concentrations of blood glucose were not different between experimental groups. Some disturbance in blood glucose concentrations were observed following gastrectomy [23]. Type of reconstructive method affects glucose level. Roux-en-Y and pouch construction and duodenal exclusion approaches were associated with high glucose levels; however, by preserving the duodenal route the pathologic glucose tolerance did not develop [31]. In a gastrectomized patient with preservation of the duodenum, a higher level of glucose was reported during the first 45 minutes after a liquid test meal [32]. Concentrations of glucose in patients with duodenal exclusion (Roux-en-Y) were higher than that of the control group; this, in turn, supports the hypothesis that exclusion of duodenal passage disturbs glucose homeostasis [33]. In the present study, the concentrations of blood glucose were in the normal range. The amount of nitrogen losses in fecal matter can be increased after gastrectomy which indicates a decrease in the quantity of ingested protein [22]. The rate of amino acid absorption in gastrectomized animals is rapid [22,25]; but the efficiency of absorption is decreased resulting in the increase of nitrogen losses through defecation. The rate of nitrogen losses in animals with a normal duodenum is less than with duodenal bypass [22].

Radiographic findings, conducted for 3 days after surgery, did not reveal any signs of anatomic abnormality, organ displacement, anastomotic leakage and narrowing or obstruction at the sites of the anastomosis. One month after surgery, radiographic findings did not show any signs of leakage or strictures at the anastomotic sites. The change in the peristaltic activity of the jejunum can lead to problematic delayed gastric emptying time. after Roux-en-Y gastrojejunostomy and occurs in 25% to 30% of patients [34]. Different types of reconstructive techniques may have different results in transit of the solid meal [7]. Gastric emptying time was less than 100 minutes and similar between experimental groups. The mean operating time was 117 ± 11.3 min in Roux-en-Y and 116 ± 16.9 min in Jejunal loop interposition [35]. In other studies in humans, mean operating time of 225 minutes for laparoscopic total gastrectomy [18] and 72 ± 4 minutes for laparoscopic assisted distal gastrectomy [17] were reported. Anastomotic leakage is one of the most important and early complications following total or partial gastrectomy, especially in esophagojejunal anastomosis because of poor blood supply to the esophagus [36]. About 35–65% of all operative and postoperative deaths are due to anastomotic breakdown and leakage [37]. In the present study, there was no sign of narrowing, stricture or obstruction in the anastomotic sites.

## Conclusion

Considering the fact that the power of the test might be low due to low number of animals in each experimental group; however, both Roux-en-Y and Jejunal loop interposition might be considered as suitable reconstructive techniques following gastrectomy with no particular difference in clinical and para-clinical parameters and postoperative complications in dogs. Further investigations with a larger number of animals and longer postoperative observation may be warranted based on the findings of the present study.

## Competing interests

The authors declare that they have no competing interests.

## Authors’ contributions

JB, MA and ARK designed and performed the study. FA conducted the biochemistry analysis. JB drafted the manuscript. ANN performed the statistical analysis and helped in interpreting the data and drafting the manuscript. All authors read and approved the final manuscript.
